# Urinary Incontinence in Men and Women After Total Hip Arthroplasty: A Retrospective Observational Cohort Study

**DOI:** 10.7759/cureus.82590

**Published:** 2025-04-19

**Authors:** Mitchell Wolden, Samantha Khanna, Cameron New, Corrina Moore, Marie K March, Edward Graham, Bijoy Thomas, Gregory C Gass, Nick Vertzyas, Sean F Mungovan

**Affiliations:** 1 Physical Therapy Program, University of Jamestown, Fargo, USA; 2 Department of Orthopaedics, The Clinical Research Institute, Westmead, AUS; 3 Department of Orthopaedics, Westmead Private Hospital, Westmead, AUS; 4 Department of Orthopaedic Surgery, St. Vincent's Private Hospital, Sydney, AUS; 5 School of Medicine, University of Notre Dame, Sydney, AUS; 6 School of Health and Biomedical Sciences, Royal Melbourne Institute of Technology (RMIT) University, Melbourne, AUS

**Keywords:** hips, total hip arthroplasty (tha), total joint arthroplasty, urinary incontinence (ui), urinary tract function

## Abstract

Background: Our understanding of improved urinary incontinence (UI) following total hip arthroplasty (THA) is incomplete. Accordingly, our aim was to investigate the association between THA (anterior and posterior approach) on UI (stress, urge, and mixed urge/stress) in older women and men presenting with UI prior to THA.

Methods: A retrospective review of 318 consecutive patients who underwent primary unilateral THA in an acute surgical hospital was undertaken. Pre- and postoperative continence status was assessed using the International Consultation of Incontinence Questionnaire Urinary Incontinence (ICIQ-UI) Short Form (ICIQ-SF).

Results: Of the 288 patients included in our study, 120 patients had preoperative UI (Incontinent Group) and 168 were continent preoperatively (Continent Group). Postoperatively, 101 patients in the Incontinent Group and 114 patients in the Continent Group completed the ICIQ-SF. Following THA, patients in the Incontinent Group had significant changes in ICIQ-SF total scores (MD=1.7; 95% CI=0.93-2.56; p<0.01), frequency scores (MD=0.7; 95% CI=0.46-0.94; p<0.01), and severity scores (MD=0.6; 95% CI=0.31-0.84; p<0.01) and a non-significant decrease in the ICIQ-SF quality of life scores (MD=0.5; 95% CI=-0.10-1.03; p<0.01). Patients' sex (p=0.94), THA surgical approach (p=0.62), and the type of UI (p=0.61) had no significant effect on postoperative ICIQ-SF total scores. There was a non-significant relationship between age (r=0.03; p=0.79) and time to postoperative follow-up visit (r=-0.11; p=0.27) with ICIQ-SF total scores. In the Continent Group, 11 patients (9.6%) had postoperative UI.

Conclusions: For women and men with UI prior to THA, there was a significant postoperative improvement in UI, independent of sex or surgical approach. Including UI assessment and clinical management strategies into the pre- and postoperative care of women and men undergoing THA procedures is indicated.

## Introduction

Total hip arthroplasty (THA) is the primary surgical procedure for the management of end-stage hip osteoarthritis, leading to significant improvements in mobility and quality of life and the resolution of pain for the majority of patients [1]. Globally, it is estimated that over one million THA surgical procedures are performed annually [2], and it is projected to increase by 280% in the United States by 2040 [3]. The greater demand for THA surgical procedures has been attributed to an increasing aging population associated with longer life expectancy and a higher prevalence of osteoarthritis [4].

For patients undergoing THA surgical procedures, pre- and postoperative physical assessments are routinely incorporated into clinical management. However, the inclusion of screening procedures for the three predominant types of urinary incontinence (UI), namely, stress, urge, and mixed [5], in pre- and postoperative physical assessment protocols is largely absent. Despite the incidence of UI in patients prior to THA (29-67%) [6,7], an incidental improvement in UI following THA has been reported (31-74%) [7,8]. Postoperative improvements in UI are associated with changes in physical function [7], hip strength [9], and pelvic floor muscle function [10].

While improvement in UI symptoms following THA is important, current research investigating UI after THA excludes preoperative data [9], does not estimate effect sizes [11], nor has examined differences between men and women [6-8,12] and the time course of postoperative improvement of UI. The types of UI (stress, urge, and mixed) have not been classified using valid and reliable outcome measures. The improvements in UI after THA surgical procedures via the anterior and posterior approaches remain unclear [7,8,11,12].

Therefore, our primary aim was to investigate the effect of THA on UI in patients with pre-existing UI prior to THA. Our secondary aims were to explore (i) how sex, surgical approach, and UI type affect postoperative outcomes and (ii) the relationship between UI changes, patient age, and timing of follow-up.

## Materials and methods

Study design

We conducted a retrospective review of medical records for consecutive patients who underwent primary unilateral THA at Westmead Private Hospital in Westmead, Australia, between September 2015 and August 2019. Ten surgeons performed primary unilateral THA at Westmead Private Hospital during the study period. To reduce the potential for confounding information, patients were excluded if they underwent (i) revision THA, (ii) primary THA with the removal of hardware, (iii) bilateral THA, and/or (iv) hemiarthroplasty THA. Given the retrospective cohort study design, a power analysis was not indicated [13]; however, to assess the statistical power of our investigation, we performed a post-hoc power analysis. Ethical approval was obtained from the Western Sydney Local Health District Human Research Ethics Committee (approval number: 2023/ETH02622). Our study followed the Strengthening the Reporting of Observational Studies in Epidemiology (STROBE) reporting guidelines [14].

Study population and outcomes

Patient demographic and clinical variables were collected from pre- and postoperative assessments. Continence status, frequency, severity, and impact of UI on quality of life were assessed using a paper format of the valid and reliable International Consultation of Incontinence Questionnaire Urinary Incontinence (ICIQ-UI) Short Form (ICIQ-SF) [15]. Patients with a preoperative ICIQ-SF score of ≥1 were categorized as the Incontinent Group, while patients with an ICIQ-SF score of 0 were assigned to the Continent Group. The type of UI was categorized as stress, urge, or mixed using patient-reported responses to items related to how and when the UI symptoms occurred. Continence status was assessed preoperatively and at three months postoperatively.

Standard postoperative patient care protocols were followed for all THA surgical procedures. Patients with a preoperative ICIQ-SF score of >1 were provided information on pelvic floor exercise and optimal bladder and gastrointestinal health on the fourth postoperative day. The information on pelvic floor exercises instructed patients on how to contract the puborectalis, bulbocavernosus, and striated urethral muscles and to perform three sets of 10 repetitions of each exercise two to three times per day. Postoperative continence status was assessed using the ICIQ-SF three months after surgery during the routine postoperative follow-up with the orthopaedic surgeon. If the ICIQ-SF was not completed at the scheduled postoperative follow-up, two attempts were made to contact the patient.

Primary Outcome

Our primary outcome was the effect size of THA on postoperative UI in the Incontinent Group, as measured by changes in the ICIQ-SF scores.

Secondary Outcomes

For the Incontinent Group, the secondary outcomes were (i) the effects of sex (men, women), THA surgical approach (anterior, posterior), and type of preoperative UI (stress, urge, mixed) on postoperative UI and (ii) the relationship between changes in UI with age and the number of days to the postoperative follow-up.

Data analysis

All data were analyzed using Stata Statistical Software: Release 17.0 (April 2021; StataCorp LLC, College Station, Texas, United States). Descriptive statistics were used to summarize patient variables. For continuous and categorical variables, an independent-samples two-tailed t-test and a two-sample test-of-proportions, respectively, were conducted to assess differences between the Incontinent and Continent Groups prior to THA.

For the Incontinent Group, a paired-samples two-tailed t-test was used to determine the mean difference (MD) in the ICIQ-SF scores (total, frequency, severity, quality of life) before and after THA. Effect size (Cohen's d) was calculated to assess the magnitude of the effect of THA on ICIQ-SF scores. Cohen's d was interpreted as a large (≥0.80), moderate (0.50-0.79), small (0.20-0.49), or negligible (≤0.19) effect [16].

Within the Incontinent Group, a two-factor-between-subject analysis of variance (ANOVA) was conducted to assess changes in ICIQ-SF scores based on the interaction of (i) sex (men, women) and time (before and after THA), (ii) surgical approach (anterior, posterior) and time (before and after THA), and (iii) type of incontinence (stress, urge, mixed stress/urge) and time (before and after THA). Omega-squared (ω2) was used to estimate effect sizes. Omega-squared was interpreted as a large (≥0.15), moderate (0.06-0.14), or small (≤0.05) effect [17]. Post-hoc analyses were performed using the Sidak adjustment method.

For the Incontinent Group, Pearson's correlation coefficient (r) was calculated to assess the relationship between changes in the ICIQ-SF scores with age and time to postoperative follow-up review. Relationships were interpreted as a large (≥0.50), moderate (0.30-0.49), small (0.10-0.29), or negligible (≤0.09) effect [16]. A p-value of less than 0.05 was considered a significant difference for all analyses.

Responder sensitivity analysis was performed with univariate logistic regression to investigate the odds of a patient completing the ICIQ-SF at the postoperative visit based on preoperative demographic variables, disability ratings, and ICIQ-SF scores.

## Results

Flow of patients through the study

**Figure 1:**
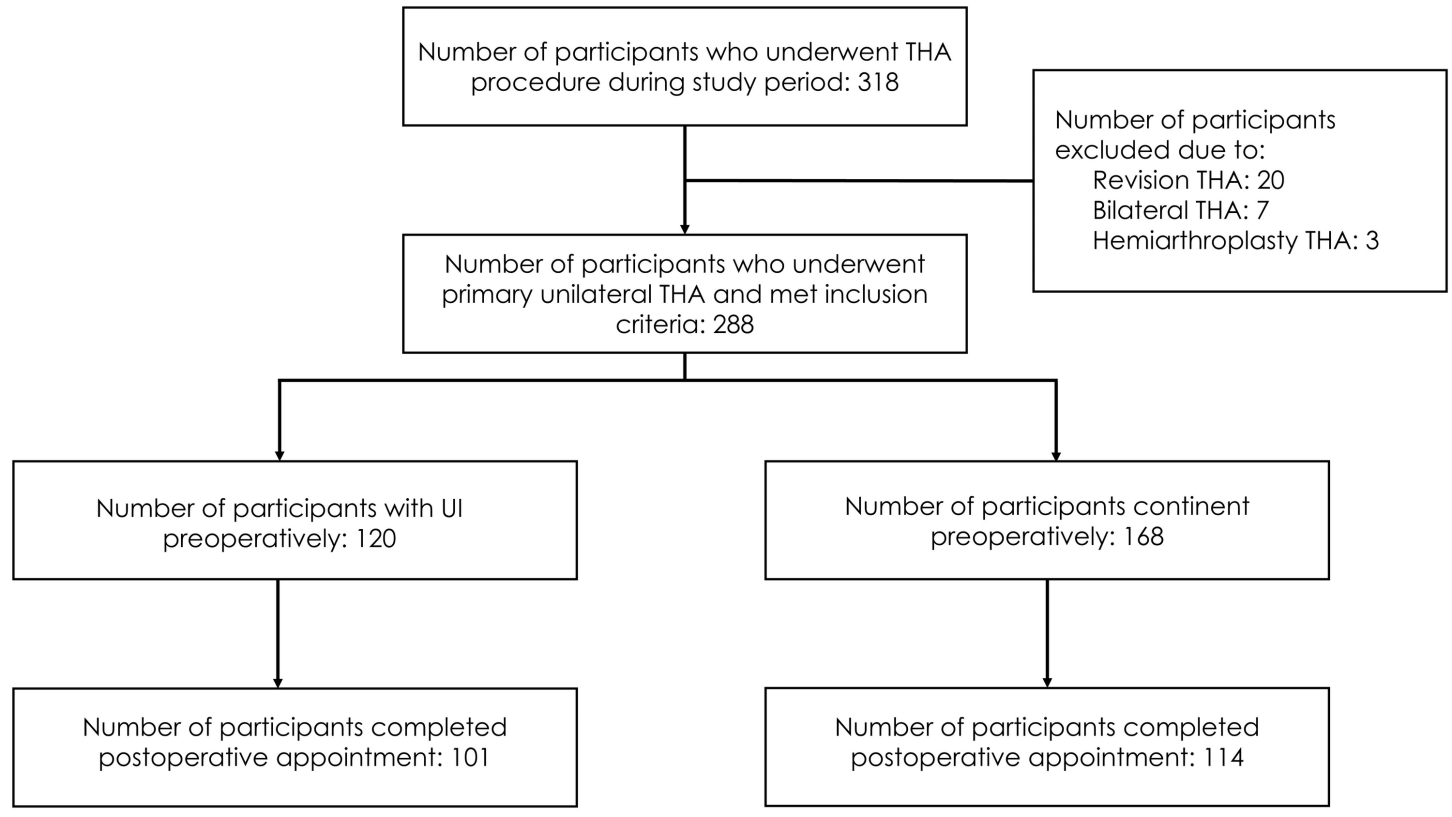
Study flow diagram THA: total hip arthroplasty; UI: urinary incontinence

A total of 318 consecutive patients underwent THA between September 2015 and August 2019. There were 288 patients who underwent THA, met the inclusion criteria, and were included in our study (Figure 1). Seventy-three patients did not complete the ICIQ-SF postoperatively. For our primary aim, post-hoc power analysis with 120 patients in the preoperative Incontinent Group and 101 patients who completed the ICIQ-SF postoperatively revealed that our study had 0.84 power.

Characteristics of the study patients

The mean age of patients (n=288) was 69.9 (SD=10.6) years; 55.9% were women, and 85.8% underwent a posterior surgical approach. The mean postoperative follow-up review was completed 110.5 (SD=34.8) days after THA. Preoperatively, 120 patients had UI and were categorized into the Incontinent Group, and 168 patients were categorized into the Continent Group. In the Incontinent Group, 54.2% of patients had preoperative urge UI. Patients in the Incontinent Group were significantly older (mean age=71.7; SD=9.6; p=0.02), had worse disability (p≤0.01-0.04), and were more likely to be women (80.8%; p<0.01) than patients in the Continent Group. There were no significant differences between the Incontinent and Continent Groups in body mass index (p=0.05), in the percentage of patients who had an anterior (p=0.72) or posterior surgical approach (p=0.72), and in the time (days) to the postoperative follow-up review (p=0.96; Table 1).

**Table 1:** Study population demographics The Incontinent Group comprised patients who had preoperative urinary incontinence, while the Continent Group comprised patients who did not have preoperative urinary incontinence, with 168 being continent preoperatively. ICIQ-SF: International Consultation of Incontinence Questionnaire Urinary Incontinence Short Form; BMI: body mass index; HOOS: Hip Disability and Osteoarthritis Outcome Score; ADL: activities of daily living; QoL: quality of life; n: sample size; SD: standard deviation; %: percentage

		Preoperative continence status		
Variable	All patients (n=288)	Incontinent (n=120)	Continent (n=168)	P-value
Age, years (mean, SD)	69.9 (10.6)	71.7 (9.6)	68.7 (11.1)	0.02
BMI (mean, SD)	30.9 (6.5)	31.8 (7.7)	30.3 (5.4)	0.05
HOOS pain (mean, SD)	41.5 (17.0)	37.9 (15.8)	44.1 (17.4)	<0.01
HOOS symptoms (mean, SD)	44.3 (19.2)	40.2 (17.9)	47.3 (19.7)	<0.01
HOOS ADL (mean, SD)	39.7 (18.2)	34.9 (15.5)	43.2 (19.3)	<0.01
HOOS sport and recreation (mean, SD)	21.3 (22.3)	17.3 (20.8)	24.2 (23.0)	0.03
HOOS hip-related QoL (mean, SD)	23.2 (19.8)	19.9 (17.6)	25.6 (21.0)	0.04
Sex, female (n, %)	161 (55.9%)	97 (80.8%)	64 (38.1%)	<0.01
Surgical approach				
Anterior approach (n, %)	41 (14.2%)	18 (15%)	23 (13.7%)	0.72
Posterior approach (n, %)	247 (85.8%)	102 (85%)	145 (86.3%)	0.74
Completed ICIQ-SF at follow-up	(n=215)	(n=101)	(n=114)	
Days to follow-up appointment (mean, SD)	110.5 (34.8)	110.6 (27.5)	110.4 (40.3)	0.96
Type of urinary incontinence		(n=120)		
Stress (n, %)		20 (16.7%)		
Urge (n, %)		65 (54.2%)		
Mixed (n, %)		35 (29.2%)		

Postoperatively, 101 patients in the Incontinent Group and 114 patients in the Continent Group completed the ICIQ-SF. In the Continent Group, 11 patients (9.6%) had postoperative UI with an average ICIQ-SF total score of 7.5 (SD=5.4). Urge incontinence was the most common type of postoperative UI in the Continent Group (n=9; 81.8%).

THA and ICIQ-SF scores

In the Incontinent Group, from preoperatively (n=120) to postoperatively (n=101), there were significant decreases in ICIQ-SF total scores (MD=1.7; 95% CI=0.93-2.56), frequency scores (MD=0.7; 95% CI=0.46-0.94), and severity scores (MD=0.6; 95% CI=0.31-0.84) and a non-significant decrease in the ICIQ-SF quality of life scores (MD=0.5; 95% CI=-0.10-1.03; Figure 1). There were a moderate effect of THA on the frequency scores (d=0.51) and a small effect on the total scores (d=0.40) and severity scores (d=0.47). There was a negligible effect on the quality of life scores (d=0.19; Table 2).

**Table 2:** Changes in urinary incontinence after THA For the Incontinent Group, a paired-samples t-test was performed. ICIQ-SF: International Consultation of Incontinence Questionnaire Urinary Incontinence Short Form; THA: total hip arthroplasty; MD: mean difference in ICIQ-SF scores from the preoperative to the postoperative visit; SD: standard deviation; 95% CI: 95% confidence interval; n: sample size; d: Cohen's d, with moderate effect size (d)=0.50-0.79, small effect size (d)=0.20-0.49, and negligible effect size (d)≤0.19

ICIQ-SF	Preoperatively (n=120), mean (SD)	Postoperatively (n=101), mean (SD)	MD (95% CI)	Effect size (d)
Total score	7.3 (4.0)	5.6 (4.6)	1.7 (0.93, 2.56)	0.40
Frequency score	2.5 (1.3)	1.8 (1.3)	0.7 (0.46, 0.94)	0.51
Severity score	2.5 (1.1)	1.9 (1.3)	0.6 (0.31, 0.84)	0.47
Quality of life score	2.4 (2.6)	1.9 (2.8)	0.5 (-0.10, 1.03)	0.19

Sex and ICIQ-SF scores

In the Incontinent Group (n=101), 83 (82.2%) women and 18 (17.8%) men completed the ICIQ-SF postoperatively. There was a small effect (ω2=0.01-0.05) with no significant difference for the interaction of sex (men, women) and time (pre- and postoperative) for all ICIQ-SF scores (Table 3). Except for the quality of life scores (p=0.33), post-hoc analyses revealed significant differences for the main effect of time (pre- and postoperative) for all other ICIQ-SF scores. There was no significant difference for the main effect of sex (men, women) (Table 3).

**Table 3:** Changes in urinary incontinence by sex Within the Incontinent Group, a two-factor-between-subject ANOVA was conducted to assess changes in ICIQ-SF scores based on the interaction of sex and time. The Sidak adjustment method was performed for post-hoc analyses. ^a^Main effect of time significant at p<0.05 ^b^Main effect of time significant at p<0.01 ICIQ-SF: International Consultation of Incontinence Questionnaire Urinary Incontinence Short Form; ANOVA: analysis of variance; THA: total hip arthroplasty; SD: standard deviation; n: sample size; ω2: omega-squared, with small effect size (ω2)≤0.05

	Preoperatively (mean, SD)		Postoperatively (mean, SD)			
ICIQ-SF	Women (n=97)	Men (n=23)	Women (n=83)	Men (n=18)	Interaction effect (p-value)	Effect size (ω2)
Total score^a^	7.4 (4.2)	6.9 (3.1)	5.6 (4.7)	5.3 (4.2)	0.94	0.03
Frequency score^b^	2.4 (1.3)	2.3 (1.1)	1.8 (1.4)	1.6 (1.1)	0.73	0.05
Severity score^a^	2.5 (1.1)	2.2 (0.8)	2.0 (1.3)	1.7 (1.2)	0.77	0.05
Quality of life score	2.4 (2.6)	2.4 (2.6)	1.9 (2.8)	2.0 (2.9)	0.88	0.01

THA surgical approach and ICIQ-SF scores

In the Incontinent Group, 12 patients (11.9%) had an anterior surgical approach, and 89 patients (88.1%) had a posterior surgical approach and completed the ICIQ-SF postoperatively. There was no significant difference for the interaction of the surgical approach (anterior, posterior) and time (pre- and postoperative) for all ICIQ-SF scores. There were a moderate effect (ω2=0.06) on the severity scores and a small effect on the total (ω2=0.03), frequency (ω2=0.05), and quality of life (ω2=0.01) scores (Table 4). Except for the quality of life scores (p=0.16), post-hoc analyses revealed significant differences for the main effect of time for all other ICIQ-SF scores. There was no significant difference for the main effect of surgical approach (anterior, posterior; Table 4).

**Table 4:** Changes in urinary incontinence by THA surgical approach Within the Incontinent Group, a two-factor-between-subject ANOVA was conducted to assess changes in ICIQ-SF scores based on the interaction of surgical approach and time. The Sidak adjustment method was performed for post-hoc analyses. ^a^Main effect of time significant at p<0.05 ^b^Main effect of time significant at p<0.01 ICIQ-SF: International Consultation of Incontinence Questionnaire Urinary Incontinence Short Form; ANOVA: analysis of variance; THA: total hip arthroplasty; SD: standard deviation; n: sample size; ω2: omega-squared, with moderate effect size (ω2)=0.06-0.14 and small effect size (ω2)≤0.05

	Preoperatively (mean, SD)		Postoperatively (mean, SD)			
ICIQ-SF	Anterior approach (n=18)	Posterior approach (n=102)	Anterior approach (n=12)	Posterior approach (n=89)	Interaction effect (p-value)	Effect size (ω2)
Total score^a^	7.1 (3.5)	7.3 (4.1)	4.6 (3.3)	5.7 (4.7)	0.62	0.03
Frequency score^b^	2.3 (1.4)	2.4 (1.2)	1.6 (1.3)	1.8 (1.4)	0.86	0.05
Severity score^a^	2.1 (0.5)	2.5 (1.2)	1.5 (0.9)	2.0 (1.3)	0.90	0.06
Quality of life score	2.6 (2.5)	2.4 (2.6)	1.5 (1.8)	1.9 (2.9)	0.52	0.01

Type of UI and ICIQ-SF scores

In the Incontinent Group (n=101), 18 (17.8%), 48 (47.5%), and 35 (34.7%) patients had stress UI, urge UI, and mixed UI, respectively. There was no significant difference for the interaction of the type of UI (stress, urge, mixed) and time (pre- and postoperative) for all ICIQ-SF scores. There were a moderate effect (ω2=0.10) on the frequency scores and a small effect on the total (ω2=0.03), severity (ω2=0.04), and quality of life scores (ω2=0.01). Except for the quality of life score (p=0.49), post-hoc analyses revealed significant differences for the main effect of time for all other ICIQ-SF scores. There was a significant difference for the main effect of type of incontinence for ICIQ-SF frequency scores. Patients with urge (MD=0.8; p<0.05) and mixed (MD=0.7; p<0.01) UI had significant decreases in their ICIQ-SF frequency scores compared to patients with stress UI (MD=0.3; p>0.05; Table 5).

**Table 5:** Changes in UI by type of incontinence Within the Incontinent Group, a two-factor-between-subject ANOVA was conducted to assess changes in ICIQ-SF scores based on the interaction of the type of UI and time. The Sidak adjustment method was performed for post-hoc analyses. ^a^Main effect of time significant at p<0.05 ^b^Main effect of time significant at p<0.01 ^c^Main effect of type of incontinence significant difference from stress incontinence at p<0.05 ^d^Main effect of type of incontinence significant difference from stress incontinence at p<0.01 ICIQ-SF: International Consultation of Incontinence Questionnaire Urinary Incontinence Short Form; UI: urinary incontinence; ANOVA: analysis of variance; THA: total hip arthroplasty; SD: standard deviation; n: sample size; ω2: omega-squared, with moderate effect size (ω2)=0.06-0.14 and small effect size (ω2)≤0.05

	Preoperatively (mean, SD)			Postoperatively (mean, SD)				
ICIQ-SF	Stress (n=20)	Urge (n=65)	Mixed (n=35)	Stress (n=18)	Urge (n=48)	Mixed (n=35)	Interaction effect (p-value)	Effect size (ω2)
Total score^a^	5.7 (3.5)	7.7 (4.2)	7.5 (3.8)	5.1 (4.0)	5.5 (5.2)	5.9 (4.0)	0.61	0.03
Frequency score^b^	1.6 (0.9)	2.5 (1.2)	2.8 (1.3)	1.3 (1.1)	1.7 (1.4)^c^	2.1 (1.3)^d^	0.55	0.10
Severity score^b^	2.4 (1.0)	2.6 (1.1)	2.3 (1.1)	1.6 (0.9)	2.0 (1.5)	2.0 (1.0)	0.52	0.04
Quality of life score	1.7 (2.1)	2.7 (2.9)	2.3 (2.2)	2.2 (2.9)	1.8 (3.0)	1.9 (2.5)	0.43	0.01

Confounding variables and ICIQ-SF scores

In the Incontinent Group, there was a non-significant and negligible relationship between age and changes in all ICIQ-SF scores (total score r=0.03 and p=0.79; frequency r=-0.04 and p=0.72; severity r=0.03 and p=0.75; quality of life r=0.04 and p=0.70). There was a non-significant and small inverse relationship between the time to a postoperative follow-up visit and changes in all ICIQ-SF scores (total score r=-0.11 and p=0.27; frequency r=-0.12 and p=0.24; severity r=-0.19 and p=0.05; quality of life r=-0.02 and p=0.87).

Responder sensitivity analysis

Univariate logistic regression analysis revealed that age (OR=1.03; 95% CI=1.00-1.05) and ICIQ-SF total scores (OR=1.10; 95% CI=1.02-1.18) were significantly associated with an increased likelihood of patients completing the postoperative visit. Undergoing the anterior surgical approach (OR=0.36; 95% CI=0.18-0.72) was significantly associated with a decreased likelihood of patients completing the postoperative visit. All other demographic variables, disability ratings, and ICIQ-SF scores had no significant relationship with completing the ICIQ-SF at the postoperative visit (Table 6).

**Table 6:** Odds of a patient completing the ICIQ-SF at the postoperative visit based on preoperative demographic variables, disability ratings, and ICIQ-SF scores Two hundred and fifteen patients completed the ICIQ-SF postoperatively; 73 patients did not complete the ICIQ-SF postoperatively. ICIQ-SF: International Consultation of Incontinence Questionnaire Urinary Incontinence Short Form; OR: odds ratio; BMI: body mass index; HOOS: Hip Disability and Osteoarthritis Outcome Score; ADL: activities of daily living; QoL: quality of life; 95% CI: 95% confidence interval

Variable	OR	95% CI	P-value
Age, years	1.03	(1.00, 1.05)	0.03
BMI	1.03	(0.99, 1.08)	0.14
Sex, female	1.52	(0.89, 2.59)	0.12
HOOS pain	0.98	(0.96, 1.00)	0.05
HOOS symptoms	0.99	(0.98, 1.01)	0.52
HOOS ADL	0.98	(0.97, 1.00)	0.06
HOOS sport and recreation	1.00	(0.98, 1.01)	0.50
HOOS hip-related QoL	1.00	(0.99, 1.02)	0.68
Surgical approach, anterior	0.36	(0.18, 0.72)	<0.01
Type of urinary incontinence			
Stress (reference category)			
Urge	0.32	(0.07, 1.53)	0.15
Mixed	1.03	(0.64, 1.44)	0.54
Preoperative ICIQ-SF scores			
Total score	1.10	(1.02, 1.18)	0.01
Frequency score	1.17	(0.78, 1.75)	0.44
Severity score	1.19	(0.71, 2.00)	0.51
Quality of life score	0.96	(0.80, 1.15)	0.67

## Discussion

To our knowledge, we are the first to investigate the magnitude of the effects of THA surgical procedures on UI. For older patients experiencing UI prior to THA, there was a low to moderate effect of THA on improving UI symptoms after THA, regardless of sex, surgical approach, or type of UI. We observed a 23% improvement in UI after THA. The inclusion of men and women, anterior and posterior surgical approaches, and all types of UI (stress, urge, and mixed) may explain the differences in our observed improvement rates of UI compared to others [7,8,12].

Sex, age, and UI

We are the first to investigate the association between THA and UI in men and women. Our study had a significantly larger proportion of patients with UI prior to THA who were women and older which is consistent with the results of others [6,18]. The higher prevalence of UI in women undergoing THA compared to men may be due to differences in internal and external sphincter muscle activation, urethral support mechanisms [19], and life events unique to women, including pregnancy, childbirth, and menopause. While UI is typically considered a women's health issue, UI significantly affects men. UI can present in 33% of men over 50 years of age [20] and up to 80% of men who undergo radical prostatectomy [21].

In our study, the Incontinent Group was significantly older than the Continent Group. For women and men, aging is associated with changes in the lower urinary tract system. Such changes include decreased maximal urethral pressure and reduced bladder function that predispose patients to an increasing risk of UI [22]. Soft tissue atrophy within the urethral sphincter complex, reduced vascularity, and decreased relative volume and density of striated muscle cells in the pelvic floor musculature have been reported as contributing factors to changes in the lower urinary tract system and UI [22].

THA surgical approach and UI

Consistent with others, the anterior and posterior surgical approaches were associated with significant postoperative improvements in UI [6]. Patients undergoing the anterior approach had a greater decrease in ICIQ-SF scores compared to patients undergoing the posterior approach. Improved UI with the anterior surgical approach may be associated with the preservation of the obturator internus muscle. The obturator internus muscle forms part of the lateral wall of the pelvis, providing stability and support to the bladder and the pelvic floor muscles, including the levator ani group (pubococcygeus, iliococcygeus, and puborectalis muscles) [6]. Our findings and the findings of others [10] were limited by the smaller number of patients undergoing the anterior approach, and responder sensitivity analysis revealed that patients undergoing the anterior surgical approach were significantly less likely to complete the ICIQ-SF postoperatively.

Stress, urge, and mixed stress/urge UI

Our study assessed the three types of UI: stress, urge, and mixed. Stress UI is the most common in women with UI [23]. Only 17% of patients in our study had stress UI. Consistent with our findings, urge UI is the most common type of UI in men [23]. To our knowledge, there has only been one study [7] that investigated changes in the three types of UI after THA. Okumura et al. reported that all types of UI improved after THA, with the greatest improvement in patients with mixed UI [7]. Consistent with the findings of Okumura et al. [7], we found improvement in the three types of UI after THA. There was a small to moderate significant improvement in patients with urge and mixed UI. The small and not statistically significant improvement for stress UI could be explained by lower ICIQ-SF total scores (5.7) prior to THA which were lower than urge (7.7) and mixed (7.5) UI.

Proposed mechanisms for improved UI after THA

Improvements in UI after THA may be attributed to increased levels of physical activity. Patients typically have less pain and a greater capacity for regular physical activity after THA [1]. Enhanced physical activity may facilitate a more timely response to the urge to urinate. Improvements in postoperative UI may be associated with increased pelvic floor muscle function, leading to increased urethral closure pressures and pelvic organ support. The pelvic floor muscles are anatomically linked to the obturator internus and adductors, which support the bladder and pelvic floor muscles, while having an important role in stabilizing the hip joint [9,10]. Significant weakness in the hip external rotators and adductors has been reported in patients with UI compared to asymptomatic individuals [9,23] and suggests a functional link between the hip and pelvic floor muscles. Strong evidence supports pelvic floor muscle training as a first-line intervention to improve UI in men and women [24]. Participation in pre- and postoperative rehabilitation programs that include hip and pelvic floor muscle strengthening after THA may further enhance the function of the obturator internus and pelvic floor muscles, contributing to better UI outcomes after THA [25].

The interactions between the central and peripheral nervous systems have been proposed as key mechanisms associated with improved postoperative UI, particularly urge UI [26]. Urge UI is associated with heightened excitability within the "brain's bladder control network" that includes the anterior cingulate cortex and the primary motor cortex [26]. Increased neural excitability of the anterior cingulate cortex and the primary motor cortex is also associated with the experience of pain [27]. THA typically leads to improvements in hip pain symptoms [28], with the reduction in pain associated with decreased excitability in the anterior cingulate and the primary motor cortex [27]. The improvements in UI for patients who present with preoperative urge UI may be associated with a reduction in the excitability of the "brain's bladder control network".

Developing UI after THA

In the Continent Group, 10% of patients reported postoperative UI. In the Incontinent Group, 21% of patients reported an increase in their UI symptoms. Our results are consistent with Okumura and colleagues who reported that 26% of patients reported an increase in UI after THA [7]. Preoperative THA patient education typically focuses on expected short-term postoperative consequences including increased pain and reduced mobility. It is unknown to what extent patients are informed of the risk of experiencing or worsening postoperative UI.

The onset or worsening of UI after THA is multifactorial. Patient mobility, medications, and indwelling urinary catheter requirements are all reported to adversely affect bladder and pelvic floor muscle function [29]. Identifying the underlying cause of UI after THA is required to develop targeted interventions, which may include pelvic floor rehabilitation, bladder retraining, medication adjustments, and addressing mobility and/or pain issues. Given the consequences of UI [30] and the prevalence of increasing UI after THA, patient education should include the risk of experiencing UI after THA. Identifying risk factors for developing or worsening UI after THA is important for the clinical management of patients undergoing THA and can facilitate the implementation of clinical protocols for prevention.

Limitations

Our sample size was 288 patients with data gathered retrospectively from one hospital. Our study population was limited to only patients who underwent primary unilateral THA and did not include those undergoing other hip arthroplasty procedures. Since our study only included patients who had one type of surgery at a single hospital, it is important to consider the possibility of selection bias that could influence our findings. While the average time to the postoperative follow-up was less than 120 days for patients with UI, the range was 62-213 days. UI was assessed using a paper questionnaire (ICIQ-SF). Patients in the Incontinent Group (16%) did not complete the ICIQ-SF after THA. An a priori power analysis was not performed to determine the sample size needed for statistical power. Postoperatively, patients were given information on pelvic floor exercises which may have influenced UI symptoms. We were unable to assess the adoption and adherence to performing pelvic floor exercises. The patients included in our study were more likely to be women and older which limit the generalizability of our findings. While appropriate for our investigation, the retrospective study design limits our ability to establish causality as we were unable to control for potential confounding variables. Further, the primary outcome of our study was ICIQ-SF scores. The ICIQ-SF is a self-report outcome measure that presents the potential for response bias from patients. Interpretation of our findings should consider the possibility of social desirability or acquiescent responses from patients. We were unable to control for potential study confounders (i.e., medications, catheter use, comorbidities) that could affect UI. Future prospective studies should investigate the potential influence that confounding variables have on UI.

Implications for clinical practice and future research

Given the prevalence of UI in patients undergoing THA (41%), preoperative screening for UI should be included in patient assessments to identify and target UI symptoms and further improve postoperative THA outcomes. Patients should be counselled on the potential for changes in UI symptoms postoperatively. There is a continued need for research to investigate the mechanisms underlying UI changes after THA and to identify patient-centered rehabilitation protocols based on surgical and patient-specific factors to maximize postoperative outcomes. UI outcomes after THA should be considered and incorporated into future economic analyses of the benefits of THA. Future investigations should also consider expanding the patient population to include other types of hip arthroplasty procedures to improve the generalizability of the findings.

## Conclusions

UI is common in women and men undergoing THA. For women and men with UI prior to THA, we report significant postoperative improvement in UI, independent of sex, surgical approach, or type of UI. However, following THA, a subset of women and men developed or experienced worsening of UI symptoms. Including UI assessment and management strategies into the pre- and postoperative care of women and men undergoing THA procedures is indicated. Research is required to objectively identify the mechanisms that contribute to improved UI after THA.
